# Supplementary material for: The impact of skeletal muscle index at the third lumbar spine on nosocomial deterioration and short-term prognosis in acute pancreatitis: a retrospective observational study
Source: PeerJ. 2024 Apr 30;12:e17283. doi: 10.7717/peerj.17283 (PMC11067894; doi:10.7717/peerj.17283)
Supplement: Supplemental Information 2 [file peerj-12-17283-s002.docx]

STROBE Statement—checklist of items that should be included in reports of observational studies

|  | Item No. | Recommendation | Page  No. | Relevant text from manuscript |
| --- | --- | --- | --- | --- |
| **Title and abstract** | 1 | (*a*) Indicate the study’s design with a commonly used term in the title or the abstract | 1 | The Impact of Skeletal Muscle Index at the Third Lumbar Spine on Nosocomial Deterioration and Short-Term Prognosis in Acute Pancreatitis: A retrospective study |
|  |  | (*b*) Provide in the abstract an informative and balanced summary of what was done and what was found | 2 | This study confirmed that diagnosing muscle depletion using L3-SMI is a valuable radiological parameter for predicting in-hospital severity and short-term prognosis in patients with acute pancreatitis. |
| Introduction | | | |  |
| Background/rationale | 2 | Explain the scientific background and rationale for the investigation being reported | 2 | Evidence confirms that the L3-SMI is a promising approach to predict hospitalization prognosis. Moreover, it's noted that a low L3-SMI constitutes an independent predictive parameter for adverse outcomes in acute pancreatitis patients. However, factors such as ethnicity, age, gender, obesity, and treatment methods influence the benchmarks for muscle depletion and malnutrition assessment using L3-SMI. |
| Objectives | 3 | State specific objectives, including any prespecified hypotheses | 2 | Regrettably, in our locale, there remains a paucity of research underscoring the prognostic value of L3-SMI in acute pancreatitis patients. This study, therefore, seeks to illuminate the relationship between the L3-SMI, as ascertained from abdominal CT scans, and the prognosis of patients with acute pancreatitis. |
| Methods | | | |  |
| Study design | 4 | Present key elements of study design early in the paper | 3 | The abdominal CT images obtained at the time of admission for each patient were retrieved. Based on the different Hounsfield values, a region of interest was segmented at the L3 level to determine the extent of the surrounding muscles, including the psoas major, erector spinae, rectus abdominis, quadratus lumborum, transverse abdominis, external and internal obliques. The average cross-sectional area of these muscles (cm2) was then calculated. Subsequently, the average cross-sectional area of the L3-level muscles (cm2) was standardized by dividing it by the square of the patient's height (m2) to calculate the L3-SMI (cm2/m2). |
| Setting | 5 | Describe the setting, locations, and relevant dates, including periods of recruitment, exposure, follow-up, and data collection | 3 | A total of 224 critically ill patients with acute pancreatitis who were admitted to Yantaishan Hospital from January 2021 to June 2022 were selected as the subjects of this study. The patients' ages ranged from 45 to 82 years, with 138 males and 86 females. Based on the in-hospital treatment outcomes, the patients were divided into two groups: a death group, consisting of 59 cases where the patients died within 7 to 30 days after admission, and a survival group, consisting of 165 cases. All samples obtained in this study were approved by the ethics committee of the Yantaishan Hospital and abided by the ethical guidelines of the Declaration of Helsinki, and ethics committee agreed to waive informed consent. |
| Participants | 6 | (*a*) *Cohort study*—Give the eligibility criteria, and the sources and methods of selection of participants. Describe methods of follow-up  *Case-control study*—Give the eligibility criteria, and the sources and methods of case ascertainment and control selection. Give the rationale for the choice of cases and controls  *Cross-sectional study*—Give the eligibility criteria, and the sources and methods of selection of participants | 3 | The inclusion criteria were as follows: ① meeting the diagnostic criteria for severe acute pancreatitis as outlined in the "China Acute Pancreatitis Diagnosis and Treatment Guidelines (2019, Shenyang)"[7]; ② complete clinical and imaging data records; ③ patient age > 18 years, with symptom onset within 3 days; ④ no anticoagulant medication taken within the past month.  The exclusion criteria were as follows: ① a history of stroke or acute myocardial infarction; ② presence of hematological disorders; ③ severe organ dysfunction such as heart, liver, kidney, lung, or concomitant tumors; ④ pregnancy or lactation in women. |
|  |  | (*b*) *Cohort study*—For matched studies, give matching criteria and number of exposed and unexposed  *Case-control study*—For matched studies, give matching criteria and the number of controls per case |  |  |
| Variables | 7 | Clearly define all outcomes, exposures, predictors, potential confounders, and effect modifiers. Give diagnostic criteria, if applicable | 3-4 | 1.2 Abdominal CT examination  Within 48 hours of admission, abdominal CT examinations were performed using a CT scanner with the following parameters: tube current of 100 mA, tube voltage of 120 kV, pitch of 1.484:1, slice thickness of 5 mm, covering a region from 2 cm above the diaphragm muscle to the anterior superior iliac spine. The procedure began with a routine plain scan, followed by the administration of a non-ionic iodinated contrast agent (Ultravist) for contrast-enhanced scanning. The injection rate was set at 2.5-3.5 mL/s, with a total volume of 80-100 mL. The arterial phase was scanned at 30-35 seconds, the portal venous phase at 60-70 seconds, and the delayed phase at 160-180 seconds. Each scan was completed during a single breath-hold. All of the aforementioned examination procedures were performed by the same physician. Two radiologists from the imaging department independently reviewed the images and conducted a comprehensive assessment.  1.3 Measurement of L3-SMI  The abdominal CT images obtained at the time of admission for each patient were retrieved. Based on the different Hounsfield values, a region of interest was segmented at the L3 level to determine the extent of the surrounding muscles, including the psoas major, erector spinae, rectus abdominis, quadratus lumborum, transverse abdominis, external and internal obliques. The average cross-sectional area of these muscles (cm2) was then calculated. Subsequently, the average cross-sectional area of the L3-level muscles (cm2) was standardized by dividing it by the square of the patient's height (m2) to calculate the L3-SMI (cm2/m2).  1.4 Modified CT Severity Index (MCTSI) and Balthazar CT Grade Assessment  The MCTSI score (ranging from 0 to 10) and the Balthazar CT grade (ranging from 0 to 4) were used for evaluation. The MCTSI score was determined based on the degree of pancreatic inflammation (0 points for normal, 2 points for pancreatitis and peripancreatic inflammation, and 4 points for the presence of fluid collection or peripancreatic fat necrosis), the extent of necrosis (0 points for no necrosis, 2 points for ≤30% necrosis, and 4 points for >30% necrosis), and the presence of extrapancreatic complications (2 points for complications such as ascites, pleural effusion, gastrointestinal or vascular involvement)[9]. The Balthazar CT grade assessment was categorized as follows: Grade A (0 points) for a normal pancreas, Grade B (1 point) for pancreatic parenchymal changes including localized or diffuse gland enlargement, Grade C (2 points) for peripancreatic and pancreatic parenchymal inflammatory changes with mild peripancreatic exudation, Grade D (3 points) for significant peripancreatic exudation or the presence of a single fluid collection within the pancreatic parenchyma or peripancreatic area, and Grade E (4 points) for extensive intra- and extra-pancreatic fluid accumulation, including pancreatic abscess, fat, and pancreatic necrosis[10]. |
| Data sources/ measurement | 8* | For each variable of interest, give sources of data and details of methods of assessment (measurement). Describe comparability of assessment methods if there is more than one group | *3-4* | *1.2 Abdominal CT examination*  *Within 48 hours of admission, abdominal CT examinations were performed using a CT scanner with the following parameters: tube current of 100 mA, tube voltage of 120 kV, pitch of 1.484:1, slice thickness of 5 mm, covering a region from 2 cm above the diaphragm muscle to the anterior superior iliac spine. The procedure began with a routine plain scan, followed by the administration of a non-ionic iodinated contrast agent (Ultravist) for contrast-enhanced scanning. The injection rate was set at 2.5-3.5 mL/s, with a total volume of 80-100 mL. The arterial phase was scanned at 30-35 seconds, the portal venous phase at 60-70 seconds, and the delayed phase at 160-180 seconds. Each scan was completed during a single breath-hold. All of the aforementioned examination procedures were performed by the same physician. Two radiologists from the imaging department independently reviewed the images and conducted a comprehensive assessment.*  *1.3 Measurement of L3-SMI*  *The abdominal CT images obtained at the time of admission for each patient were retrieved. Based on the different Hounsfield values, a region of interest was segmented at the L3 level to determine the extent of the surrounding muscles, including the psoas major, erector spinae, rectus abdominis, quadratus lumborum, transverse abdominis, external and internal obliques. The average cross-sectional area of these muscles (cm2) was then calculated. Subsequently, the average cross-sectional area of the L3-level muscles (cm2) was standardized by dividing it by the square of the patient's height (m2) to calculate the L3-SMI (cm2/m2).*  *1.4 Modified CT Severity Index (MCTSI) and Balthazar CT Grade Assessment*  *The MCTSI score (ranging from 0 to 10) and the Balthazar CT grade (ranging from 0 to 4) were used for evaluation. The MCTSI score was determined based on the degree of pancreatic inflammation (0 points for normal, 2 points for pancreatitis and peripancreatic inflammation, and 4 points for the presence of fluid collection or peripancreatic fat necrosis), the extent of necrosis (0 points for no necrosis, 2 points for ≤30% necrosis, and 4 points for >30% necrosis), and the presence of extrapancreatic complications (2 points for complications such as ascites, pleural effusion, gastrointestinal or vascular involvement)[9]. The Balthazar CT grade assessment was categorized as follows: Grade A (0 points) for a normal pancreas, Grade B (1 point) for pancreatic parenchymal changes including localized or diffuse gland enlargement, Grade C (2 points) for peripancreatic and pancreatic parenchymal inflammatory changes with mild peripancreatic exudation, Grade D (3 points) for significant peripancreatic exudation or the presence of a single fluid collection within the pancreatic parenchyma or peripancreatic area, and Grade E (4 points) for extensive intra- and extra-pancreatic fluid accumulation, including pancreatic abscess, fat, and pancreatic necrosis[10].* |
| Bias | 9 | Describe any efforts to address potential sources of bias | 4 | The statistical analysis was carried out with the aid of SPSS 25.0 software. Numerical data were tested for normality, which was confirmed using a normality test. Continuous variables were reported as mean ± standard deviation. Then, the group differences were further assessed using the t-test. Categorical variables were showed as frequencies as well as its percentages. Then, its group differences were analyzed using the chi-square test. The prognostic value of L3-SMI for acute pancreatitis was evaluated using receiver operating characteristic (ROC) curve analysis, with comparison of the area under the curve (AUC) performed using the Z-test. Pearson's correlation test was applied to analyze the correlation between variables. Multivariable logistic regression analysis was run to identify factors influencing the prognosis of acute pancreatitis. The doorway for statistical significance was established at P < 0.05. |
| Study size | 10 | Explain how the study size was arrived at | 3 | A total of 224 critically ill patients with acute pancreatitis who were admitted to Yantaishan Hospital from January 2021 to June 2022 were selected as the subjects of this study. |

Continued on next page

| Quantitative variables | 11 | Explain how quantitative variables were handled in the analyses. If applicable, describe which groupings were chosen and why | 3-4 | 1.2 Abdominal CT examination  Within 48 hours of admission, abdominal CT examinations were performed using a CT scanner with the following parameters: tube current of 100 mA, tube voltage of 120 kV, pitch of 1.484:1, slice thickness of 5 mm, covering a region from 2 cm above the diaphragm muscle to the anterior superior iliac spine. The procedure began with a routine plain scan, followed by the administration of a non-ionic iodinated contrast agent (Ultravist) for contrast-enhanced scanning. The injection rate was set at 2.5-3.5 mL/s, with a total volume of 80-100 mL. The arterial phase was scanned at 30-35 seconds, the portal venous phase at 60-70 seconds, and the delayed phase at 160-180 seconds. Each scan was completed during a single breath-hold. All of the aforementioned examination procedures were performed by the same physician. Two radiologists from the imaging department independently reviewed the images and conducted a comprehensive assessment.  1.3 Measurement of L3-SMI  The abdominal CT images obtained at the time of admission for each patient were retrieved. Based on the different Hounsfield values, a region of interest was segmented at the L3 level to determine the extent of the surrounding muscles, including the psoas major, erector spinae, rectus abdominis, quadratus lumborum, transverse abdominis, external and internal obliques. The average cross-sectional area of these muscles (cm2) was then calculated. Subsequently, the average cross-sectional area of the L3-level muscles (cm2) was standardized by dividing it by the square of the patient's height (m2) to calculate the L3-SMI (cm2/m2).  1.4 Modified CT Severity Index (MCTSI) and Balthazar CT Grade Assessment  The MCTSI score (ranging from 0 to 10) and the Balthazar CT grade (ranging from 0 to 4) were used for evaluation. The MCTSI score was determined based on the degree of pancreatic inflammation (0 points for normal, 2 points for pancreatitis and peripancreatic inflammation, and 4 points for the presence of fluid collection or peripancreatic fat necrosis), the extent of necrosis (0 points for no necrosis, 2 points for ≤30% necrosis, and 4 points for >30% necrosis), and the presence of extrapancreatic complications (2 points for complications such as ascites, pleural effusion, gastrointestinal or vascular involvement)[9]. The Balthazar CT grade assessment was categorized as follows: Grade A (0 points) for a normal pancreas, Grade B (1 point) for pancreatic parenchymal changes including localized or diffuse gland enlargement, Grade C (2 points) for peripancreatic and pancreatic parenchymal inflammatory changes with mild peripancreatic exudation, Grade D (3 points) for significant peripancreatic exudation or the presence of a single fluid collection within the pancreatic parenchyma or peripancreatic area, and Grade E (4 points) for extensive intra- and extra-pancreatic fluid accumulation, including pancreatic abscess, fat, and pancreatic necrosis[10]. |
| --- | --- | --- | --- | --- |
| Statistical methods | 12 | (*a*) Describe all statistical methods, including those used to control for confounding | 4 | The statistical analysis was carried out with the aid of SPSS 25.0 software. Numerical data were tested for normality, which was confirmed using a normality test. Continuous variables were reported as mean ± standard deviation. Then, the group differences were further assessed using the t-test. Categorical variables were showed as frequencies as well as its percentages. Then, its group differences were analyzed using the chi-square test. The prognostic value of L3-SMI for acute pancreatitis was evaluated using receiver operating characteristic (ROC) curve analysis, with comparison of the area under the curve (AUC) performed using the Z-test. Pearson's correlation test was applied to analyze the correlation between variables. Multivariable logistic regression analysis was run to identify factors influencing the prognosis of acute pancreatitis. The doorway for statistical significance was established at P < 0.05. |
|  |  | (*b*) Describe any methods used to examine subgroups and interactions | 4 | The statistical analysis was carried out with the aid of SPSS 25.0 software. Numerical data were tested for normality, which was confirmed using a normality test. Continuous variables were reported as mean ± standard deviation. Then, the group differences were further assessed using the t-test. Categorical variables were showed as frequencies as well as its percentages. Then, its group differences were analyzed using the chi-square test. The prognostic value of L3-SMI for acute pancreatitis was evaluated using receiver operating characteristic (ROC) curve analysis, with comparison of the area under the curve (AUC) performed using the Z-test. Pearson's correlation test was applied to analyze the correlation between variables. Multivariable logistic regression analysis was run to identify factors influencing the prognosis of acute pancreatitis. The doorway for statistical significance was established at P < 0.05. |
|  |  | (*c*) Explain how missing data were addressed | N/A | N/A |
|  |  | (*d*) *Cohort study*—If applicable, explain how loss to follow-up was addressed  *Case-control study*—If applicable, explain how matching of cases and controls was addressed  *Cross-sectional study*—If applicable, describe analytical methods taking account of sampling strategy | N/A | N/A |
|  |  | (*e*) Describe any sensitivity analyses | N/A | N/A |
| Results | | | | |
| Participants | 13* | (a) Report numbers of individuals at each stage of study—eg numbers potentially eligible, examined for eligibility, confirmed eligible, included in the study, completing follow-up, and analysed | 4-5 | There were no statistically significant differences (P > 0.05) between the survivor as well as non-survivor groups in terms of sex, age, BMI, etiology, duration of anti-inflammatory drug use, and proportion of surgical patients. However, the non-survivor group had significantly higher admission scores for APACHE II, proportion of mechanically ventilated patients, and proportion of patients receiving renal replacement therapy compared to the survivor group (P < 0.05). (Table 1) |
|  |  | (b) Give reasons for non-participation at each stage | N/A | N/A |
|  |  | (c) Consider use of a flow diagram | N/A | N/A |
| Descriptive data | 14* | (a) Give characteristics of study participants (eg demographic, clinical, social) and information on exposures and potential confounders | 4-5 | Table 1 |
|  |  | (b) Indicate number of participants with missing data for each variable of interest | N/A | N/A |
|  |  | (c) *Cohort study*—Summarise follow-up time (eg, average and total amount) | N/A | N/A |
| Outcome data | 15* | *Cohort study*—Report numbers of outcome events or summary measures over time | N/A | N/A |
|  |  | *Case-control study—*Report numbers in each exposure category, or summary measures of exposure | *5* | *The non-survivor group had significantly higher MCTSI scores (6.42±0.69), Balthazar CT grades (3.78±0.45) compared to the survivor group (P < 0.05). Conversely, the non-survivor group had a lower L3-SMI (39.68±3.25) compared to the survivor group (42.71±4.28), and this difference was statistically significant (P < 0.05). (Table 2)* |
|  |  | *Cross-sectional study—*Report numbers of outcome events or summary measures | N/A | N/A |
| Main results | 16 | (*a*) Give unadjusted estimates and, if applicable, confounder-adjusted estimates and their precision (eg, 95% confidence interval). Make clear which confounders were adjusted for and why they were included | Figure 1 | Figure 1 |
|  |  | (*b*) Report category boundaries when continuous variables were categorized | 5 | In the logistic regression analysis, with the mortality of patients with acute pancreatitis as the dependent variable, MCTSI score, Balthazar CT grade, L3-SMI, admission APACHE II score, mechanical ventilation, and renal replacement therapy were considered as independent variables. The results indicated that all the aforementioned factors were significant risk factors for mortality in patients with acute pancreatitis (P < 0.05). (Table 5) |
|  |  | (*c*) If relevant, consider translating estimates of relative risk into absolute risk for a meaningful time period | 5 | In the logistic regression analysis, with the mortality of patients with acute pancreatitis as the dependent variable, MCTSI score, Balthazar CT grade, L3-SMI, admission APACHE II score, mechanical ventilation, and renal replacement therapy were considered as independent variables. The results indicated that all the aforementioned factors were significant risk factors for mortality in patients with acute pancreatitis (P < 0.05). (Table 5) |

Continued on next page

| Other analyses | 17 | Report other analyses done—eg analyses of subgroups and interactions, and sensitivity analyses | N/A | N/A |
| --- | --- | --- | --- | --- |
| Discussion | | | | |
| Key results | 18 | Summarise key results with reference to study objectives | 6 | this study unveils that L3-SMI serves as a mortality risk factor for patients with acute pancreatitis. Receiver operating characteristic curve analysis reveals that the area under the curve for poor prognosis in terms of L3-SMI is 0.816, signifying high diagnostic value. The optimal cut-off value discerned for L3-SMI is 0.348, with an associated sensitivity of 0.667 and specificity of 0.941. The reason for this stems from the understanding that acute pancreatitis triggers inflammation of the pancreas, leading to subsequent hemorrhagic necrosis. This often escalates into a concomitant infection of pancreatic and peripancreatic necrotic tissues[16]. In the absence of effective infection control, this can culminate in pancreatic abscess, severe systemic infection, sepsis, metabolic aberrations, gastrointestinal dysfunction, and widespread organ damage[16]. |
| Limitations | 19 | Discuss limitations of the study, taking into account sources of potential bias or imprecision. Discuss both direction and magnitude of any potential bias | 7-8 | However, this study has some limitations. As a single-center retrospective study conducted at Yantaishan Hospital, the generalizability of the results may be limited. A multicenter study with a larger sample size would help validate the findings. Additionally, potential confounding factors such as smoking status, alcohol use, and medication history were not fully adjusted for in the analysis. Nutritional status is a dynamic process, but L3-SMI was only measured at admission and changes over the course of treatment were not assessed. Other markers of nutritional status and inflammation such as albumin, prealbumin, and C-reactive protein levels were not investigated. Finally, long-term outcomes beyond the in-hospital period were not evaluated. Further prospective studies with more comprehensive assessment of potential prognostic factors are needed to strengthen the conclusions regarding the role of L3-SMI in predicting outcomes in acute pancreatitis. |
| Interpretation | 20 | Give a cautious overall interpretation of results considering objectives, limitations, multiplicity of analyses, results from similar studies, and other relevant evidence | 7 | The APACHE II scoring system, hinging on clinical and biochemical parameters, has demonstrated proficiency in evaluating acute pancreatitis severity and prognosis. However, its complexity and intricate operationalization pose constraints[21]. In contrast, the Balthazar CT grading system promotes timely identification of acute pancreatitis etiology while faithfully reflecting the extent of pancreatic and peripancreatic hemorrhage and necrosis. Based on these, patients can be categorized into five tiers (0-4)[22]. Clinical investigations have indicated that higher Balthazar CT grades correlate with more severe acute pancreatitis and elevated patient mortality rates, an observation consistent with this analysis. Thus, Balthazar CT grading exhibits a correlation with the severity of acute pancreatitis, and treatment strategies can be calibrated based on this grading and other indicators to eschew misjudgments and delayed disease progression[23]. |
| Generalisability | 21 | Discuss the generalisability (external validity) of the study results | 7 | The study discovered that deceased patients had higher MCTSI scores and Balthazar CT grades than surviving patients, showing statistically significant differences. L3-SMI levels were lower in the deceased group compared to the survival group, with a statistically significant difference. Furthermore, there was a negative correlation between L3-SMI and the MCTSI score and the Balthazar CT grade, suggesting that L3-SMI, similar to the MCTSI score and Balthazar CT grade, can be utilized in assessing the severity and short-term prognosis of acute pancreatitis in a hospital setting. |
| Other information | |  | | |
| Funding | 22 | Give the source of funding and the role of the funders for the present study and, if applicable, for the original study on which the present article is based | N/A | N/A |

*Give information separately for cases and controls in case-control studies and, if applicable, for exposed and unexposed groups in cohort and cross-sectional studies.

**Note:** An Explanation and Elaboration article discusses each checklist item and gives methodological background and published examples of transparent reporting. The STROBE checklist is best used in conjunction with this article (freely available on the Web sites of PLoS Medicine at http://www.plosmedicine.org/, Annals of Internal Medicine at http://www.annals.org/, and Epidemiology at http://www.epidem.com/). Information on the STROBE Initiative is available at www.strobe-statement.org.
