# Supplementary material for: The impact of skeletal muscle index at the third lumbar spine on nosocomial deterioration and short-term prognosis in acute pancreatitis: a retrospective observational study
Source: PeerJ. 2024 Apr 30;12:e17283. doi: 10.7717/peerj.17283 (PMC11067894; doi:10.7717/peerj.17283)
Supplement: Supplemental Information 3 [file peerj-12-17283-s003.docx]

|  | **0** | **1** | **2** | **3** |
| --- | --- | --- | --- | --- |
| Gender | Female | Male | - | - |
| Surgery | Negative | Positive | - | - |
| Mechanical Ventilation | Negative | Positive | - | - |
| Renal Replacement Therapy | Negative | Positive | - | - |
| Etiology | - | Cholangiogenic | Alcoholic | Hyperlipidemic |
